# Multi-phase contrast-enhanced magnetic resonance image-based radiomics-combined machine learning reveals microscopic ultra-early hepatocellular carcinoma lesions

**DOI:** 10.1007/s00259-022-05742-8

**Published:** 2022-03-01

**Authors:** Kui Sun, Liting Shi, Jianfeng Qiu, Yuteng Pan, Ximing Wang, Haiyan Wang

**Affiliations:** 1 Department of Radiology, Shandong Provincial Hospital Affiliated to Shandong First Medical University, Jing Wu Road, No. 324, Jinan, 250021 China; 2 Department of Radiology, Shandong First Medical University & Shandong Academy of Medical Sciences, Taian, 271016 China; 3 Medical Science and Technology Innovation Center, Shandong First Medical University & Shandong Academy of Medical Sciences, Jinan, 250000 China

**Keywords:** Radiomics, Magnetic resonance imaging, Hepatocellular carcinoma, Machine learning

## Abstract

**Purpose:**

This study aimed to investigate whether models built from radiomics features based on multiphase contrast-enhanced MRI can identify microscopic pre-hepatocellular carcinoma lesions.

**Methods:**

We retrospectively studied 54 small hepatocellular carcinoma (SHCC, diameter < 2 cm) patients and 70 patients with hepatocellular cysts or haemangiomas from September 2018 to June 2021. For the former, two MRI scans were collected within 12 months of each other; the 2^nd^ scan was used to confirm the diagnosis. The volumes of interest (VOIs), including SHCCs and normal liver tissues, were delineated on the 2^nd^ scans, mapped to the 1^st^ scans via image registration, and enrolled into the SHCC and internal-control cohorts, respectively, while those of normal liver tissues from patients with hepatocellular cysts or haemangioma were enrolled in the external-control cohort. We extracted 1132 radiomics features from each VOI and analysed their discriminability between the SHCC and internal-control cohorts for intra-group classification and the SHCC and external-control cohorts for inter-group classification. Five radial basis-function, kernel-based support vector machine (SVM) models (four corresponding single-phase models and one integrated from the four-phase MR images) were established.

**Results:**

Among the 124 subjects, the multiphase models yielded better performance on the testing set for intra-group and inter-group classification, with areas under the receiver operating characteristic curves of 0.93 (95% CI, 0.85–1.00) and 0.97 (95% CI, 0.92–1.00), accuracies of 86.67% and 94.12%, sensitivities of 87.50% and 94.12%, and specificities of 85.71% and 94.12%, respectively.

**Conclusion:**

The combined multiphase MRI-based radiomics feature model revealed microscopic pre-hepatocellular carcinoma lesions.

**Supplementary Information:**

The online version contains supplementary material available at 10.1007/s00259-022-05742-8.

## Introduction

According to the World Cancer Report published by the International Agency for Research on Cancer (IARC), hepatocellular carcinoma (HCC), the third leading cause of cancer-related mortality worldwide, accounts for approximately 80% of primary liver cancers. In 2020, there were an estimated 410,038 new cases and 391,152 deaths from HCC in China [1]. Cirrhosis secondary to hepatitis B virus (HBV) infection is a major risk factor for HCC and affects the majority of middle-aged and elderly Chinese men [2]. Additional main risk factors for HCC observed worldwide include external exposure to toxins (aflatoxin exposure, alcohol consumption) and hepatitis C virus (HCV) infection. However, the early manifestations of the process of HCC development from dysplastic nodules (DNs) to small hepatocellular carcinoma (SHCC), which is defined by the very early HCC criteria of the Barcelona Clinic Liver Cancer (BCLC) criteria as a single HCC nodule less than 2 cm in diameter, are difficult to detect clinically. Thus, a large number of Chinese patients are diagnosed at an advanced stage and receive only palliative care [3]. The 5-year survival rate for HCC is only 33% in all races [4], but in China, it drops to 14.1% [5]. Therefore, it is specifically essential to find a new measurement to detect these changes in the early stage for diagnosis and intervention.

Medical imaging technologies, including contrast-enhanced computed tomography (CE-CT), contrast-enhanced magnetic resonance imaging (CE-MRI), and contrast-enhanced ultrasound imaging (CE-US), can achieve satisfactory diagnostic results for HCC. If the imaging profile on dynamic MR is specific for HCC (intense contrast-enhanced agent uptake in the arterial phase followed by extracellular contrast wash-out in the venous and/or delayed phase), a diagnosis can be made even without histological confirmation [6–8]. However, it is still difficult to identify very early lesions by medical imaging, even for an experienced radiologist. Although these lesions may have already become malignant, they remain microscopic on dynamic MRI and lack typical radiological markers [9].

The term “radiomics” was first proposed by Lambin et al. [10]. It is a field that focuses on improving image analysis and the high-throughput extraction of a vast number of quantitative features from medical images. The underlying basis of radiomics is that the analysis of these quantitative features can provide more and better information than physicians can by visually analysing these images. It has been reported that radiomics features of the tumour area present significant predictive efficacy in the classification of HCC [11–15]. Li Yang et al. [16] and Huang et al. [17] showed the best areas under the receiver operating characteristic curve (AUROCs) of 0.861 and 0.784 in the validation cohort, respectively. However, these previous studies were conducted on the assumption that HCC lesions could be observed on MR images, and few studies have focused on early or very early HCC lesions with no imaging changes.

Hence, we aimed to develop a multiphase MRI-based radiomics model to evaluate the risk of microscopic pre-HCC lesions for HCC and/or other liver-related disease patients.

## Materials and methods

### Patient cohort

The institutional review board of our institution approved this retrospective study. The requirement for informed consent was waived.

From September 2018 to June 2021, we retrieved the data of hospitalized patients screened for HCC due to cirrhosis secondary to HBV infection from Shandong Provincial Hospital affiliated to Shandong First Medical University.

**Fig. 1:**
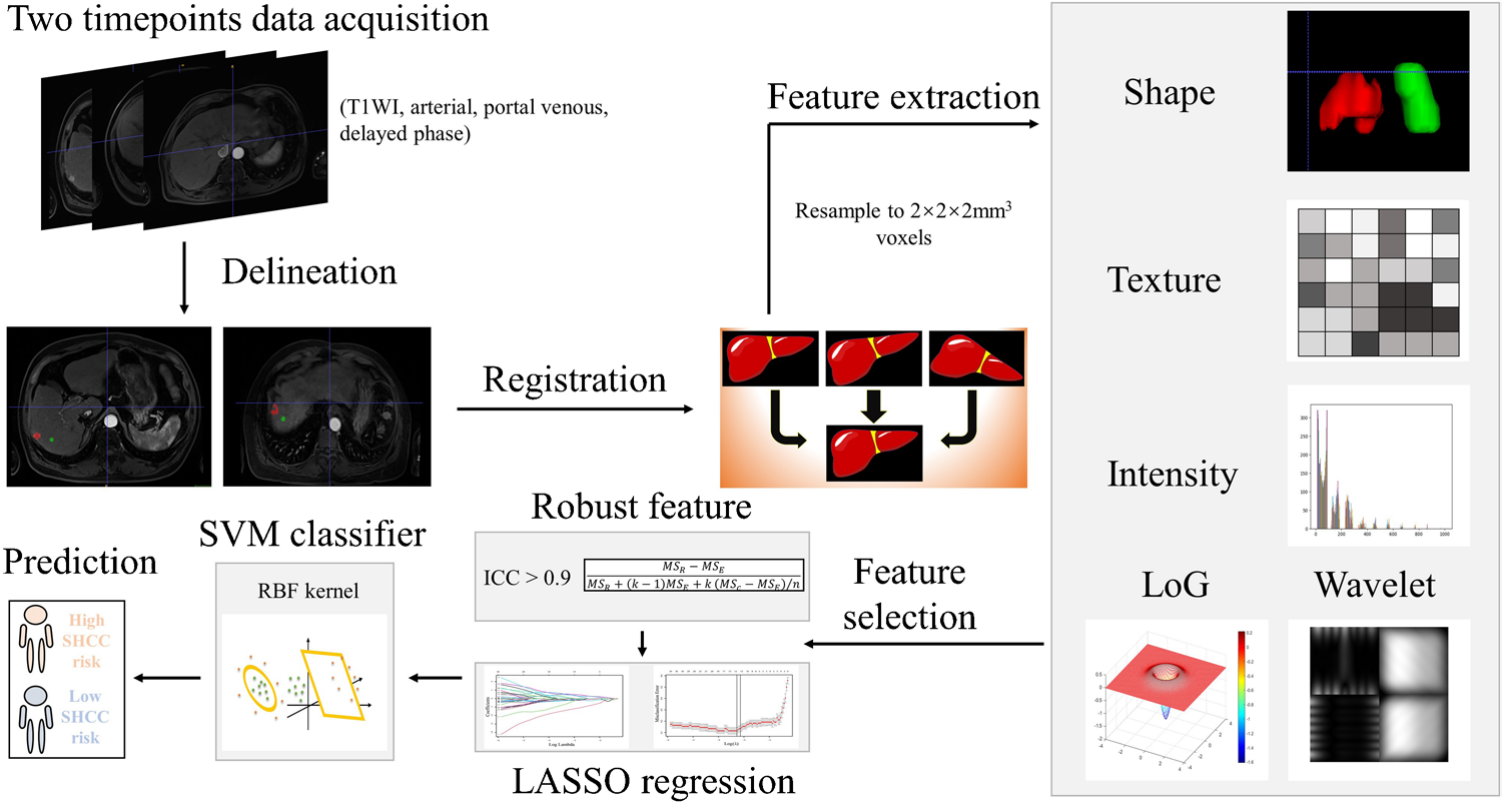
The flow chart of the whole experiment includes several steps of data acquisition, registration, outlining, feature extraction and selection, model construction, and prediction

The inclusion criteria for SHCC patients were as follows: (1) patients were diagnosed with SHCC by a radiologist with more than ten years of experience. (2) Patients received two MR examinations, of which SHCC was not apparent on the former but was diagnosed on the latter. The interval between the two examinations was not more than one year. (3) Patients had never been treated with transcatheter arterial chemoembolization (TACE) in the newly developed SHCC lesion. (4) Patients’ MR imaging at the newly developed SHCC lesion site was consistent with the ‘wash-in and wash-out’ phenomenon described in the guidelines. (5) Patients had complete T1-weighted, arterial-phase, portal venous–phase, and delayed-phase MR images at both timepoints. The SHCC lesions on the first MR scan of eligible SHCC patients were included in the SHCC cohort; a normal volume of interest (VOI) from the first MR scan of each eligible SHCC patient was included in the internal-control cohort. Moreover, we also set up an external-control cohort to ensure the robustness of the research findings. The inclusion criteria are as follows: (1) patients were diagnosed with hepatic cyst or haemangioma by a radiologist with more than ten years of experience. (2) There were one to three lesions, and each one measured less than 2.3 cm in diameter. (3) Patients had no history of hepatitis, drug-induced liver damage, or alcohol abuse. (4) Patients had complete T1-weighted, arterial-phase, portal venous–phase, and delayed-phase MR images. The exclusion criteria were as follows: (1) the tumour outline was unclear on MR images in the SHCC cohort. (2) The quality of the MR images was poor in the internal- or external-control cohort. Figure 1 shows the whole experimental design.

### MR image acquisition and registration and delineation of volumes of interest (VOIs)

The MR image acquisition parameters are detailed in Table 1. The MR images were retrieved from the picture archiving and communication (PACS) system, including non-contrast-enhanced T1-weighted (T1WI), arterial phase, portal venous phase, and delayed phase images. In patients with SHCC, we collected two MR scans within an interval of less than 12 months. Imaging characteristics consistent with SHCC lesions could be detected in the 2^nd^ scan but no visible changes could be observed by the naked eye in the exact lesion location in the 1^st^ scan.

**Table 1:** The detail of the MR image acquisition

Parameters			
Scanner	Siemens 3.0 T Skyra (TIM Systems, Siemens Medical Solutions, Erlangen, Germany)	Siemens 3.0 T Prisma (TIM Systems, Siemens Medical Solutions, Erlangen, Germany)	Siemens 3.0 T Verio (TIM Systems, Siemens Medical Solutions, Erlangen, Germany)
TR (ms)	4.31 (median)	3.92 (median)	3.92 (median)
TE (ms)	2.03 (median)	1.39 (median)	1.39 (median)
Slice Thickness (mm)	3 (median)	3 (median)	3 (median)
Dose of Gd-EOB-DTPA MR contrast agent	0.1 mmol/kg	13 ml	13 ml
Injection rate of Gd-EOB-DTPA MR contrast agent	2.0 ml/s	2.5 ml/s	2.5 ml/s
Acquisition time of the arterial phase	14 s	19 s	19 s
Acquisition time of the portal venous phase	26–30 s	25–32 s	60 s
Acquisition time of the delayed phase	60 s	3–5 min	3–5 min

To ensure accurate VOI delineation, we used the Elastix toolbox [18] in the 3D slicer open-source software [19] version 4.1.1 (http://www.slicer.org) to register the images successively. The arterial phase images of the 2^nd^ scan were used as a template, and the arterial and other phases of the 1^st^ scan were the targets to be successively registered.

Then, a radiologist with 20 years of experience manually delineated the SHCC tumour VOIs on the template images using ITK-SNAP open-source software version 3.8.0 (Yushkevich P and Gerig G). The tumour delineation covered the entire SHCC tumour lesion in all slices. For the internal-control cohort, the radiologist delineated the normal liver VOIs of these patients on MR images. A normal liver VOI was defined as a lack of imaging abnormality changes associated with SHCC on ten successive levels on both scans. The VOI for the external-control cohort was delineated identically as for the internal-control cohort. Consequently, we obtained 68 SHCC VOIs, 54 internal-control VOIs, and 70 external-control VOIs on the MR images from each phase.

### Feature extraction

We performed a rescaling operation to normalize the MR images. The resampled voxel sizes were set to 2 × 2 × 2 mm^3^ to standardize the slice thickness. We mapped the VOIs onto the registered image to extract radiomics features using the PyRadiomics package [20] version 3.0.1. The radiomics features were generated from the original, wavelet-filtered, and Laplacian of Gaussian (LoG)-filtered images. The features included shape, intensity (‘First-order statistics’), and texture. Texture features included grey-level cooccurrence matrix (GLCM), grey-level size zone matrix (GLSZM), grey-level run length matrix (GLRLM), neighbouring grey-tone difference matrix (NGTDM), and grey-level dependence matrix (GLDM) features.

### Feature robustness and reproducibility

The robustness and reproducibility of the features were assessed with the intraclass correlation coefficient (ICC) [21–23]. Thirty patients from the SHCC cohorts were chosen randomly for VOI re-delineation by another experienced radiologist two weeks after the first delineation. Features with an ICC coefficient greater than 0.9 were retained and considered to have excellent robustness and reproducibility. Moreover, we concatenated the four-phase MR image features to evaluate whether the joint-phase (all-phase) features provided better discriminability than the single-phase features.

### Feature selection

We implemented two types of analysis: intra-group classification and inter-group classification. The intra-group classification involved the 68 SHCC VOIs and 54 internal-control VOIs, and the inter-group classification involved the 68 SHCC VOIs and 70 external-control VOIs.

We randomly divided the intra-group classification (*n* = 122) and the inter-group classification (*n* = 138) data into a training set (*n* = 92 for intra-group classification, *n* = 104 for inter-group classification) and testing set (*n* = 30 for intra-group classification, *n* = 34 for inter-group classification) at a 3:1 ratio. These datasets came from the four sets of single-phase features and the single set of all-phase features. In total, there were ten datasets.

The least absolute shrinkage and selection operator (LASSO) regression model was used for feature selection. Employing regularization, the LASSO regression model adjusts the penalty coefficient value (*λ*), compresses most of the coefficients to zero, and retains the values with nonzero coefficients. Consequently, the retained features are nonredundant and sparse, potentially preventing the classifier from overfitting. By adjusting the parameter (*λ*) with the training set, the optimal features were screened via the minimum criteria with tenfold cross-validation.

### Classifier and assessment of the performance of different models

We chose the radial basis-function, kernel-based support vector machine (RBF-SVM) as the classifier. First, the most valuable features filtered by the LASSO regression model from the four single phase-based and single combined-phase MR image features were used to train the corresponding classification models with the training set (intra-group and inter-group classification). The optimal parameters of the SVM models were selected via tenfold cross-validation. Second, the efficacy of all models was tested with the testing set (intra-group and inter-group classification). Five models were established based on the RBF-SVM classifier: four according to the individual image phases (T1WI, arterial phase, portal venous phase, and delayed phase), and an all-phase model according to the integration of the four single-phase MR image features. The area under the receiver operating characteristic curve (AUROC), area under the precision-recall curve (AUPRC), sensitivity, specificity, and accuracy were calculated to evaluate classifier performance.

### Statistical analysis

The SPSS version 26.0 (http://www.ibm.com/, IBM) and R version 4.0.1 (https://www.r-project.org/) statistical software was used for statistical analysis. The chi-square test was used to assess categorical data. The Shapiro–Wilk test was used to assess the normality of continuous data. The *t* test was used if the continuous data conformed to a normal distribution; otherwise, the Mann–Whitney *U* test was used. Normally distributed data are described as the mean ± SD; otherwise, the median (IQR) was used. Differences in AUC values between different models on the testing set were estimated using the Delong test. A two-sided *p* < 0.05 was regarded as significant.

## Results

### Baseline characteristics

A total of 124 patients were enrolled in this study, including 54 SHCC patients (ranging from 36 to 73 years of age, with a mean of 56.9 ± (9.35) years) and 70 hepatic cyst or haemangioma patients (ranging from 36 to 73 years of age, with a mean of 48.5 ± (14.88) years). In the SHCC group, 4 patients were women and 50 were men. In the hepatic cyst or haemangioma group, 30 patients were women and 40 were men. There were statistically significant differences in age (*p* < 0.001 [*t* value: 3.65]) and gender (*p* < 0.001 [χ^2^ value: 16.99]) between the two groups. The interval between the two MRI scans was 4.81 ± (2.67) months. Details of the VOI numbers, volumes, and diameters are provided in supplementary Table 2.

### Optimal radiomics signature

We extracted 1132 radiomics features from the VOIs at each phase, consisting of 14 shape, 18 intensity, 68 texture, 688 wavelet, and 344 LoG features. Through the first round of screening using the ICC, 179, 325, 277, and 266 features remained among the T1WI and arterial, portal venous, and delayed phase image features, respectively (Fig. 1a of the supplementary material).

Subsequently, after filtration with LASSO regression, the T1WI, arterial phase, portal venous phase, delayed phase, and all-phase models were built with 9, 6, 6, 23, and 36 optimal radiomics features, respectively (Fig. 1b1, b2 of the supplementary material). The retained features of each model were considered the radiomics signatures in the intra-group classification. The all-phase model consisted of 8 T1W and 12 arterial phase, 8 portal venous phase, and 8 delayed phase image features (Fig. 1c1 of the supplementary material).

In addition, 16, 13, 12, 10, and 18 optimal radiomics features (Fig. 1b3, b4 of the supplementary material) were selected by LASSO regression in the models built from the T1WI, arterial phase, portal venous phase, delayed phase, and all-phase imaging features in the inter-group classification, respectively. The all-phase model was constructed from 7 T1W, 3 arterial phase, 1 portal venous phase, and 7 delayed phase imaging features (Fig. 1c2 of the supplementary material). The optimal radiomics signature of each model is detailed in Table 1 of the supplementary material.

### Best performance of the models in intra-group and inter-group classification

**Fig. 2:**
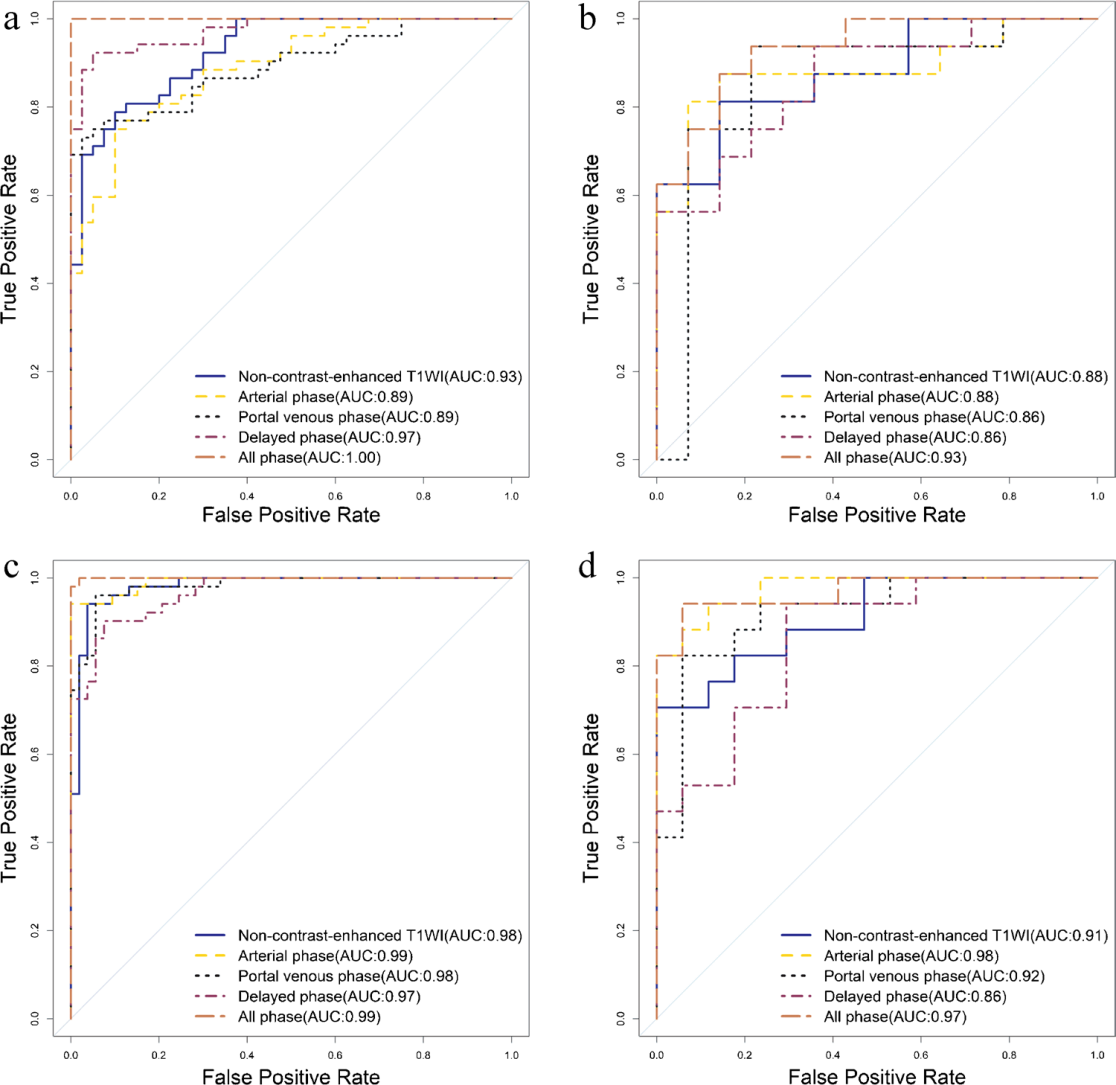
Comparison of the AUROC on the training set (**a**) and testing set (**b**) of the different models from the intra-group classification, and training set (**c**), testing set (**d**) from the inter-group classification

**Fig. 3:**
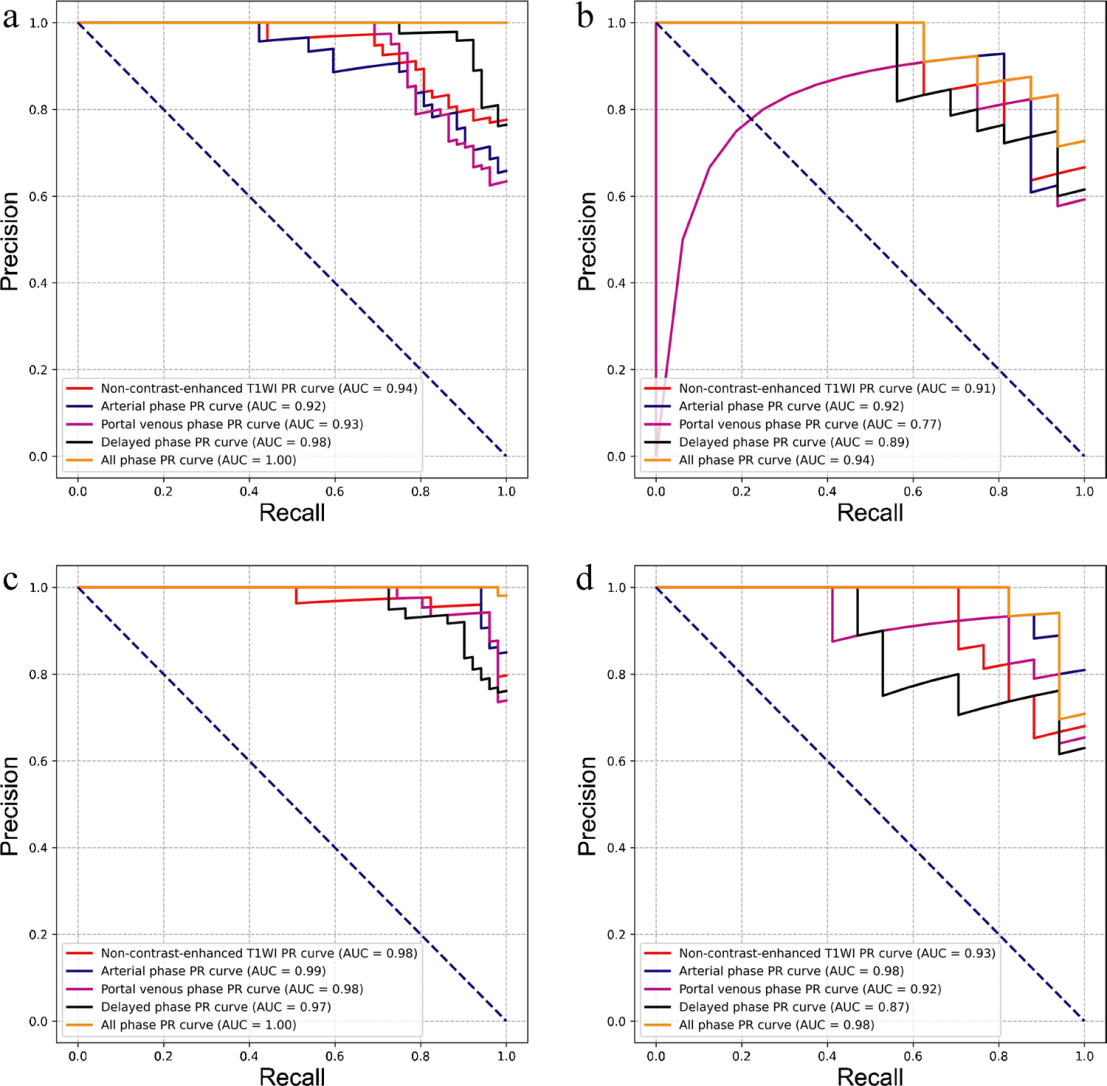
Comparison of the AUPR on the training set (**a**) and testing set (**b**) of the different models from the intra-group classification, and training set (**c**), testing set (**d**) from the inter-group classification

For intra-group classification, the performance of the all-phase model was significantly greater than that of the single-phase classifiers. The optimal performance was achieved with an AUROC of 1.00 (95% CI, 1.00–1.00) with the training set and 0.93 (95% CI, 0.85–1.00) with the testing set (Fig. 2) and corresponding AUPRCs of 1.00 and 0.94 (Fig. 3), respectively. Basic first-order statistics (‘Range’ and ‘Maximum’) and high-dimensional texture features (Grey-Level Co-occurrence Matrix [‘DifferenceAverage’] and Grey-Level Size Zone Matrix [‘ZoneVariance’]) contributed to the model construction.

Likewise, the all-phase model achieved good performance in inter-group classification with the 18 optimal radiomics features. The RBF-SVM classifier achieved an AUROC of 0.99 (0.99–1.00) with the training set and 0.97 (95% CI, 0.92–1.00) on the testing set (Fig. 2) and corresponding AUPRCs of 1.00 and 0.98 (Fig. 3), respectively. In model construction, the most important contributing features were first-order statistics (‘Maximum’ and ‘Variance’) and high-dimensional texture features (Grey-Level Size Zone Matrix [‘ZoneVariance’] and Grey-Level Dependence Matrix [‘HighGrayLevelEmphasis’]). The performance details of the different models are shown in Table 2.

**Table 2:** The performance details of different models

Group									
Data set	Intra-					Inter-			
	Model	AUC (95%CI)	Acc	Sen	Spe	AUC (95%CI)	Acc	Sen	Spe
Training set	T1WI	0.93(0.877–0.976)	83.70%	78.85%	90.00%	0.98(0.958–1)	95.19%	94.12%	96.23%
	Arterial phase	0.89(0.815–0.951)	81.52%	75.00%	90.00%	0.99(0.981–1)	97.12%	94.12%	100%
	Portal venous phase	0.89(0.829–0.957)	83.70%	73.08%	97.50%	0.98(0.961–1)	95.19%	96.08%	94.34%
	Delayed phase	0.97(0.946–0.999)	93.48%	92.31%	95.00%	0.97(0.939–0.994)	91.35%	90.20%	92.45%
	All-phase	1 (1–1)	100%	100%	100%	0.99(0.999–1)	99.04%	100%	98.11%
Testing set	T1WI	0.88(0.759–1)	83.33%	81.25%	85.71%	0.91(0.816–1)	85.29%	70.59%	100%
	Arterial phase	0.88(0.754–1)	86.67%	81.25%	92.86%	0.98(0.937–1)	91.18%	82.35%	100%
	Portal venous phase	0.86(0.695–1)	86.67%	93.75%	78.57%	0.92(0.827–1)	88.24%	82.35%	94.12%
	Delayed phase	0.86(0.731–0.992)	80.00%	93.75%	64.29%	0.86(0.740–0.983)	82.35%	94.12%	70.59%
	All-phase	0.93(0.85–1)	86.67%	87.50%	85.71%	0.97(0.916–1)	94.12%	94.12%	94.12%

Abbreviation: *intra*-, intra-group classification; *inter*-, inter-group classification; *CI*, confident interval; *AUC*, the area under the curve; *Acc*, accuracy; *Sen*, sensitivity; *Spe*, specificity

### Differences in the AUROCs between intra-group and inter-group classification

**Fig. 4:**
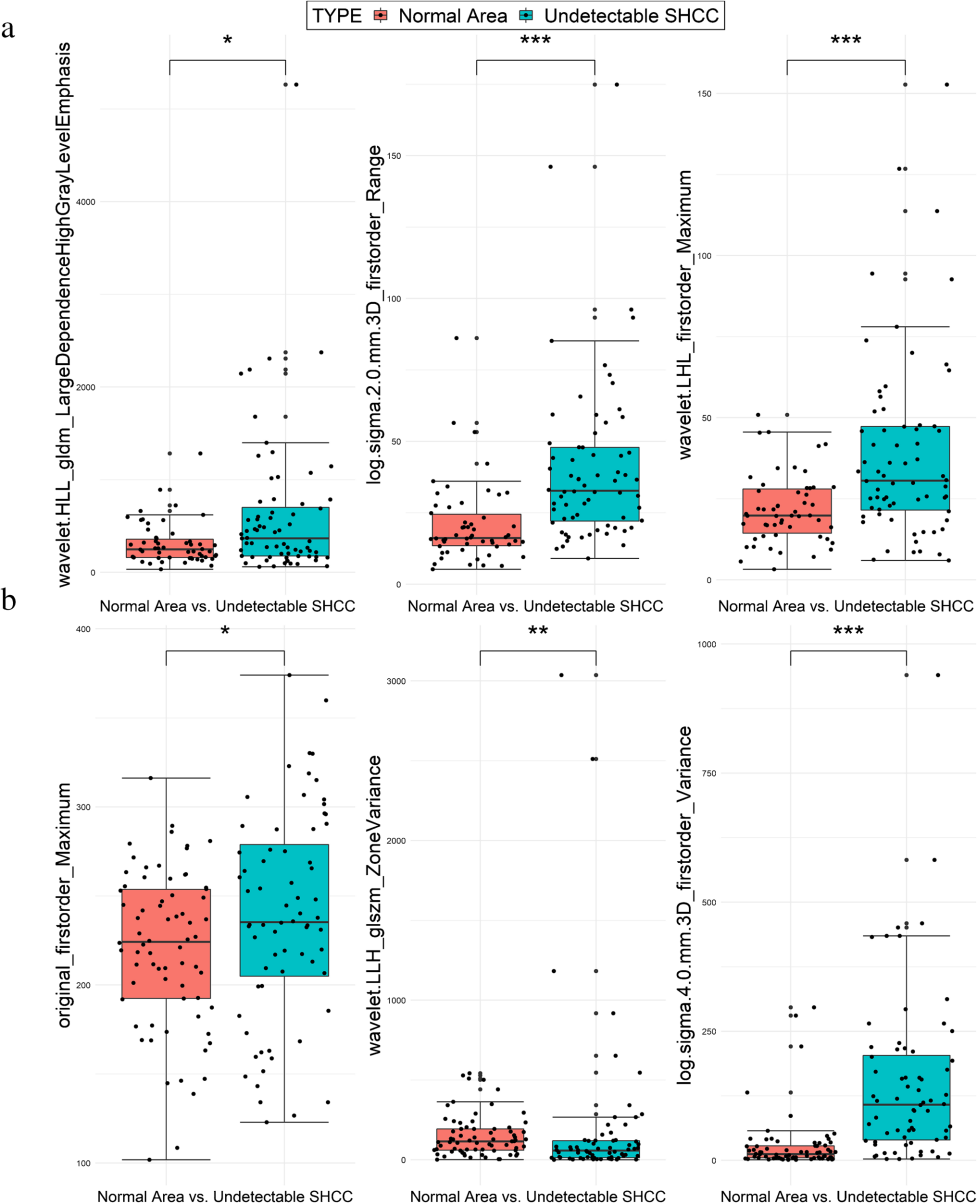
Statistical analysis of the top 3 radiomics features a weight of intra-group classification (**a**) and inter-group classification (**b**) on the all-phase model

**Fig. 5:**
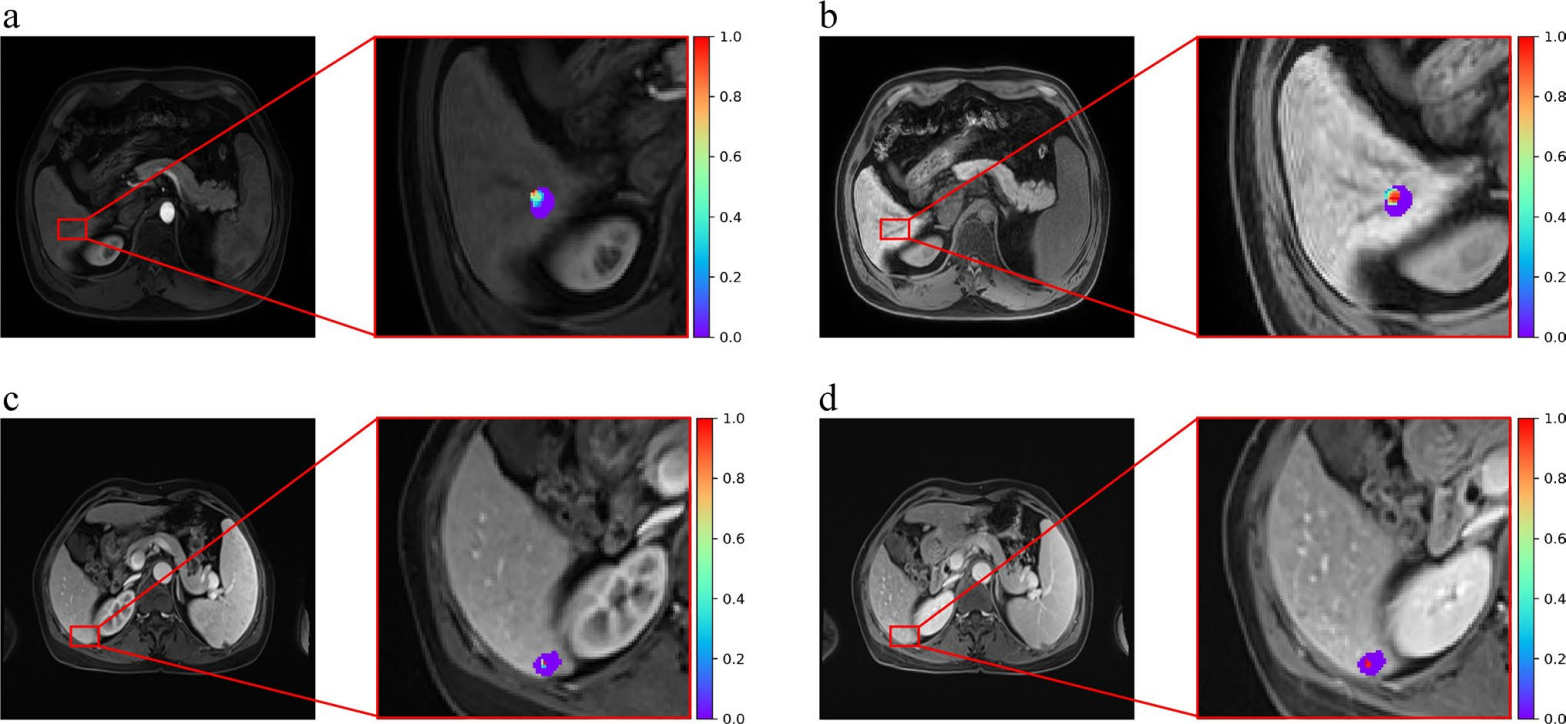
Feature visualization of the gray level dependence matrix (GLDM) of arterial phase MRI by wavelet filter (**a**). Feature visualization of the first order Range of T1WI phase MRI by Laplacian of Gaussian (LoG; *σ* = 2 mm) filter (**b**). Feature visualization of the gray level size zone matrix (GLSZM) of arterial phase MRI by wavelet filter (c). Feature visualization of the first order Maximum of delayed phase MRI by none filter (**d**)

With the testing set, we compared the performance of the same model in intra-group and inter-group classification according to the AUROC values. The Delong test revealed no differences between the values for the two classifications, *p* > 0.05 (Table 3). In addition, the top 3 radiomics features from the weighted ranking of the all-phase model were analysed to explore whether there were significant differences between normal tissue and undetectable SHCC lesions (Table 4). The results showed a significant difference (*p* < 0.05) in the values of the radiomics features between normal tissue area and undetectable SHCC lesions (Fig. 4). Figure 5 depicts expression heatmaps for the top two weighted features of the all-phase model for intra-group and inter-group classification. The heatmaps reveal that the heterogeneity within the tumour cannot be reflected by MRI images, increasing the interpretability of the radiomics features.

**Table 3:** Comparison of the AUROC between inter- and intra-group classification on the testing set

Model name (inter- vs. intra-)	AUROC values	*p* values
T1W1 vs. T1W1	0.91 vs. 0.88	0.698
Arterial phase vs. arterial phase	0.98 vs. 0.88	0.194
Portal venous phase vs. portal venous phase	0.92 vs. 0.86	0.510
Delayed phase vs. delayed phase	0.86 vs. 0.86	0.999
All-phase vs. all-phase	0.97 vs. 0.93	0.478

Abbreviations: *inter*-, inter-group classification; *intra*-, intra-group classification; *AUROC*, the area under the receiver operator characteristic curve

**Table 4:** The top 3 radiomics features weight of inter- and intra-group classification on all-phase model

Model	Filter	Feature class	Feature	Image phase	Weight
All-phase model (intra-)	Wavelet (HLL)	GLDM	Large Dependence High Gray Level Emphasis	Arterial	11.0103
	LoG (*σ* = 2 mm)	First order	Range	T1WI	9.6618
	Wavelet (LHL)	First order	Maximum	Delayed	7.9649
All-phase model (inter-)	Original	First order	Maximum	Delayed	7.2267
	Wavelet (LLH)	GLSZM	Zone Variance	Arterial	3.8969
	LoG (*σ* = 4 mm)	First order	Variance	Delayed	2.8917

Abbreviations: *intra*-, intra-group classification; *inter*-, inter-group classification; *LoG*, Laplacian of Gaussian; *GLDM*, gray level dependence matrix; *GLSZM*, gray level size zone matrix

## Discussion

In this study, we aimed to develop a multiphase MRI-based radiomics model to evaluate the risk of microscopic lesions or high-risk nodules in HCC patients with radiologically undetectable lesions. This model showed clearly excellent performance in both intra-group and inter-group classification. These results are clinically significant in that such models can help physicians find potential malignant lesions for HCC and/or other liver-related disease patients in the very early stage.

In a majority of Chinese HCC patients, the disease tends to be secondary to HBV infection [24, 25], and a large proportion of patients are generally identified at an advanced stage [26]. Cirrhosis increases the risk of SHCC/HCC [27]. The transformation of cirrhotic nodules into HCC is a continuous and complex process [28], following the development of low-grade dysplastic nodules (LGDNs) to high-grade dysplastic nodules (HGDNs) to HCC [29]. Among them, DN is considered to be a precancerous lesion of SHCC/HCC [30–35]. DNs usually present with nontypical manifestations of influence on gadoxetic acid (GD-EoB-DTPA)-enhanced MRI [36, 37]. According to a study by Eremites SC et al. [38], LGDNs result in only a 4% increase in arterial blood supply, HGDNs, an approximately 17–32% increase, and HCC, an approximately 94% increase. Therefore, a large proportion of DNs presents with uniform T1 and T2 signals on MRI and does not show enhancement in the arterial phase [39], which makes it difficult to distinguish [40, 41]. The diagnosis of SHCC on MR depends on the increase in unmatched arterioles, the decrease in blood supply to the portal vein, the deposition of iron and lipids, and the changes in the formation of envelopes. From our experience, even before the radiologist can recognize SHCC/HCC lesions on MRI by the naked eye, the precancerous lesion may already exist. During the development of DNs or SHCC, few clinical symptoms are present, which can be fatal. Therefore, early inspection, detection, intervention, and treatment are particularly important.

To date, there have been several radiomics studies on hepatocellular carcinoma. Li Yang et al. [16] achieved an AUROC of 0.861 with the validation cohort of an MRI-based model incorporating significant clinical radiological factors and a fusion radiomics signature obtained from hepatobiliary phase (HBP) images. Huang et al. [17] reported that radiomics feature models based on CE-MR images had favourable performance in predicting HCC, with mean AUROCs of 0.712, 0.784, 0.771, and 0.774 when constructed from arterial phase, portal venous phase, delayed phase, and hepatobiliary phase features, respectively. Many studies have been based on analyses of lesions that have already changed on visual imaging [42–47]. This makes sense if the lesion is detected before it can be observed by the naked eye.

In the current study, we included both an internal-control and an external-control cohort. If we had only set up an internal-control cohort, there could have been biases in the experimental results because we would not have been able to guarantee that the selected normal VOIs would not be disturbed by the information from underlying malignant lesions (although we attempted to avoid this as much as possible). Hence, we also selected a group of normal liver VOIs from patients diagnosed with hepatic haemangiomas or cysts as the external-control cohort to ensure a more stable experiment. The experimental results also confirm the existence of biases in one aspect; the AUROC for inter-group classification was generally better than that for intra-group classification with the testing set. However, this difference was not significant, with *p* > 0.05 obtained with the Delong test. This result indicates that despite the existence of biases, these models can still exert a predictive efficacy on the corresponding populations. The goal of the intra-group classification analysis is to screen HCC patients early to determine whether new high-risk lesions have developed, while the purpose of inter-group classification analysis is to determine whether there are risky lesions related to HCC in the screening of non-HCC populations. We invited two radiologists (Mj.X. and Lq.C.) with five years of experience to perform a reader test with the testing set (Fig. 2 of the supplementary material). In intra-group classification, the AUROCs obtained by the two radiologists were 0.62 and 0.75, respectively. In the inter-group classification, the AUROCs obtained by the two radiologists were 0.71 and 0.73, respectively. When lesions are still in the microscopic stage, very early diagnosis can be greatly challenging for radiologists. Our model can assist radiologists in diagnosing these lesions ultra-early, producing great benefit to the patients.

Radiomics features related to image transformation are highly important for revealing information on very small lesions. The most common such transformation is the application of filters, as the image information obtained by different filter transformations may be different. According to Zhang et al. [48], the AUROC of a model constructed from filter-free radiomics features was 0.728 with the validation cohort. In a study of Zhao et al. [45], the radiomics features extracted from images processed by the wavelet filter were not included; only the original features and the features extracted from images transformed by the LoG filter were included. Among them, most of the features for constructing the optimal model were LoG features, comprising approximately ~ 75%, and the AUROC was 0.771 with the validation cohort. Throughout our research, in the best models for intra-group and inter-group classification, the features extracted from images subjected to LoG filtering and wavelet transformation accounted for ~ 97% and ~ 78% of the model construction, and the AUROCs were 0.93 and 0.97 with the testing set, respectively. The information contained in the original images is not sufficient to explain all the phenomena and meet clinical needs. For further image analysis and research, some edge detection methods are often applied, such as LoG filters and wavelet transforms. The LoG is composed of a Gaussian kernel and Laplacian kernel; the latter is sensitive to areas with rapidly changing intensities, highlighting specific texture information in the original texture image and enhancing edges. A Gaussian smoothing filter is usually needed to smooth the image before the Laplacian operation to reduce the susceptibility to noise. Wavelet transformation produces good local characteristics. When the scale of the wavelet function is large, the anti-noise ability is strong, and when the scale of the wavelet function is small, the ability to extract image details is strong. Therefore, a balance between suppressing noise and extracting image edge details can be achieved. We recognized that some features reflecting tumour heterogeneity and microenvironment [7] were intensity (first-order) and texture (GLCM, GLSZM, etc.) features, consistent with other studies [22, 49–52]. Depending on the organ being imaged and the type of imaging modality, the first-order statistics may or may not have been the same across all applications. In intra-group classification, the top three features used to construct the best model were ‘Large Dependence High Grey Level’, ‘Range’, and ‘Maximum’ (Table 4). We found that the median values of these features in the lesion areas were generally higher than that in the normal areas [367.65 (175.13, 712.37) vs. 247.59 (159.17, 364.83), 32.73 (21.89, 47.89) vs. 16.17 (13.31, 24.88), and 30.61 (21.39, 47.29) vs. 19.83 (13.81, 28.25)]. In inter-group classification, the top three features used to construct the best model were ‘Maximum’, ‘Zone Variance’, and ‘Variance’ (Table 4). The average value of the ‘Maximum’ feature of the lesion areas was greater than that of the normal area [238.23 ± 58.92 vs. 220.63 ± 44.07]. The median of the ‘Variance’ feature value of the lesion areas was greater than that of the normal area [107.86 (38.94, 205.54) vs. 12.99 (5.01, 28.06)], but the ‘Zone Variance’ value was lower than that of the normal area [57.72 (59.56, 193.61) vs. 115.79 (12.76, 119.92)]. ‘Entropy’ and ‘Uniformity’ are two commonly used features computed in medical imaging. In a liver study [53], researchers showed that the ‘Total Entropy’ of the liver of healthy people was higher than that of patients with liver metastases. We calculated ‘Sum Entropy’ instead of ‘Total Entropy’ and found that the results corresponded to the above conclusions [1.39 (1.17, 1.64) vs. 1.44 (1.38, 1.50)]. Griethuysen et al. [18] stated that ‘Zone Entropy’ measures the uncertainty/randomness in the distribution of zone sizes and grey levels. The higher values obtained in the lesion indicates more heterogeneity [3.41 (2.58, 4.05) vs. 2.00 (1.58, 2.58)] than in the normal area.

## Limitations

The limitations of this study are as follows: first, the contour of the tumour area relied on manual delineation by an experienced radiologist, which required considerable time and energy expenditures. Second, this study relies on powerful registration algorithms, which can directly affect the accuracy of segmentation. For this reason, we asked the experienced radiologists to double-check the registration effect to minimize the impact of registration uncertainty on the experimental results. Third, a small number of samples were included, and this was a single-centre, retrospective study. The results of this study thus reflect only the patients at the centre and are not representative of the general population. Therefore, multicentre, large sample, and prospective studies are needed to further improve the results of this study.

Indeed, the essence of our experiment is hindsight. When applied to reality, we cannot define the VOI on the first MRI scan because there are no abnormalities when observed by the naked eye. However, the essence of research is to solve practical problems. The clinical transformations of this research in the future could entail the following: first, we need a software algorithm to automatically or semiautomatically identify and segment the liver. Second, a sliding convolution kernel of a specific size, e.g., 5 × 5 or 7 × 7, would extract features from left to right sequentially for the whole liver or a specific liver segment and input these quantitative data into our model to obtain the predicted probability. These probabilities can be visualized with a heatmap, which conveniently displayed which areas are at high risk. This could help clinicians make decisions and intervene to achieve precision medicine.

## Conclusions

Although new lesions in SHCC patients cannot be observed on MR imaging, a combination of radiomics features and machine learning algorithms can be sensitive to underlying abnormalities that cannot be detected by the naked eye.

In our study, we reported that the optimal model, based on the integration of radiomics features from four phases of MR images, could achieve excellent performance in evaluating microscopic pre-hepatocellular carcinoma lesions.

## Supplementary Information

Below is the link to the electronic supplementary material.

Supplementary file1 (DOCX 735 KB)

## Data Availability

The datasets generated during the current study are available from the corresponding author on reasonable request.
