# Supplementary material for: Multi-phase contrast-enhanced magnetic resonance image-based radiomics-combined machine learning reveals microscopic ultra-early hepatocellular carcinoma lesions
Source: Eur J Nucl Med Mol Imaging. 2022 Mar 1;49(8):2917–28. doi: 10.1007/s00259-022-05742-8 (PMC9206604; doi:10.1007/s00259-022-05742-8)
Supplement: Supplementary file 1 — Supplementary file1 (DOCX 735 KB) [file 259_2022_5742_MOESM1_ESM.docx]

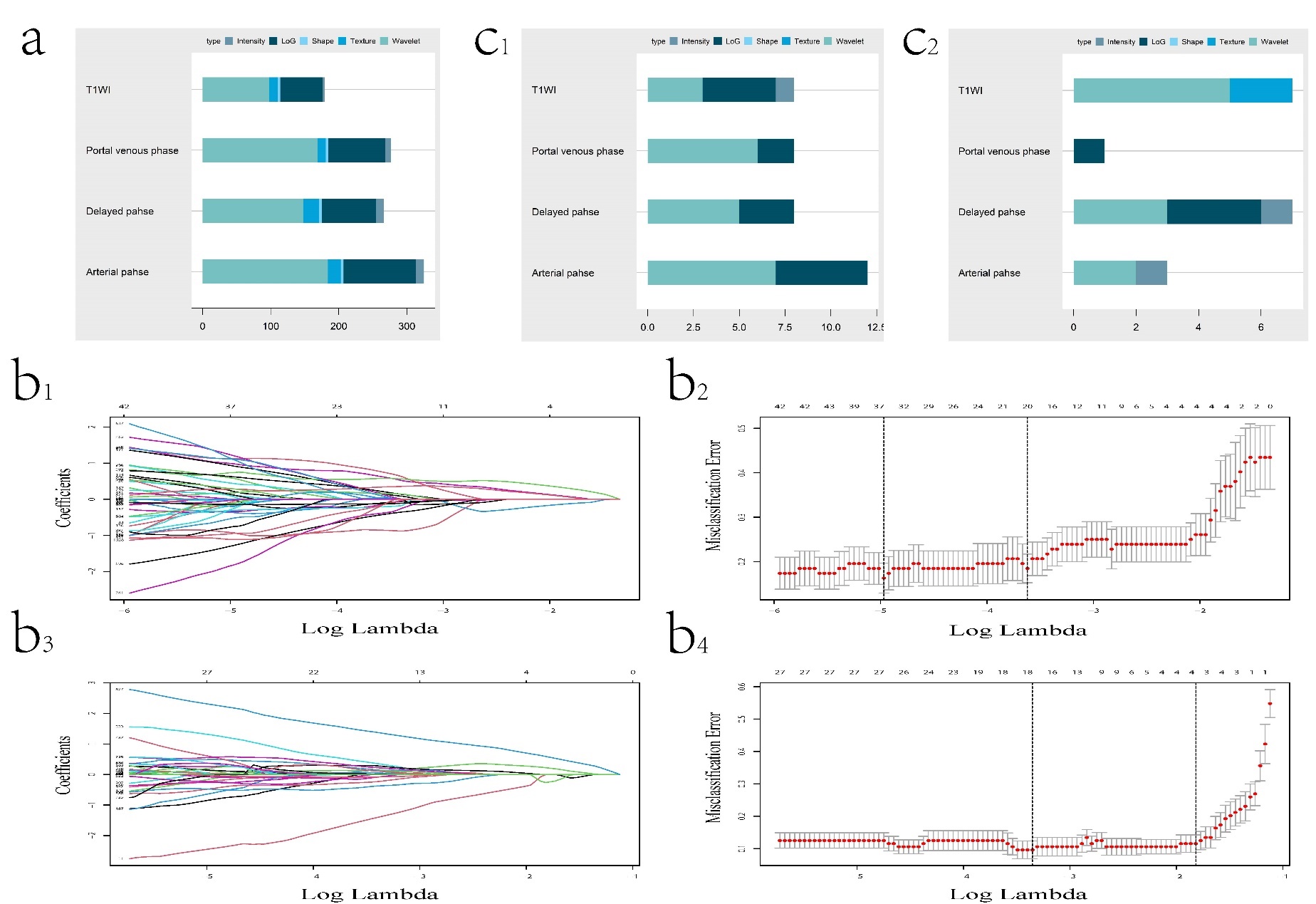


Figure1. The proportion of the robust radiomics feature on each MR phase after ICC, LASSO coefficient graph of the optimal All-phase model on the intra-group classification and inter-group classification, and the proportion of the optimal radiomics signature on each MR phase of the All-phase model on intra-group classification and inter-group classification. The proportion of the robust radiomics feature on each MR phase after ICC (a). The LASSO coefficient graph of the optimal All-phase model on the intra-group classification (b1) and inter-group classification (b3). The LASSO diagram shows that the optimal features are selected by the parameter (λ) via ten-fold cross-validation and the minimum criteria. The first vertical line indicates the best features for the All-phase model on intra-group classification (b2) and inter-group classification (b4). The proportion of the optimal radiomics signature on each MR phase of the all-phase model on intra-group classification (c1) and inter-group classification (c2).


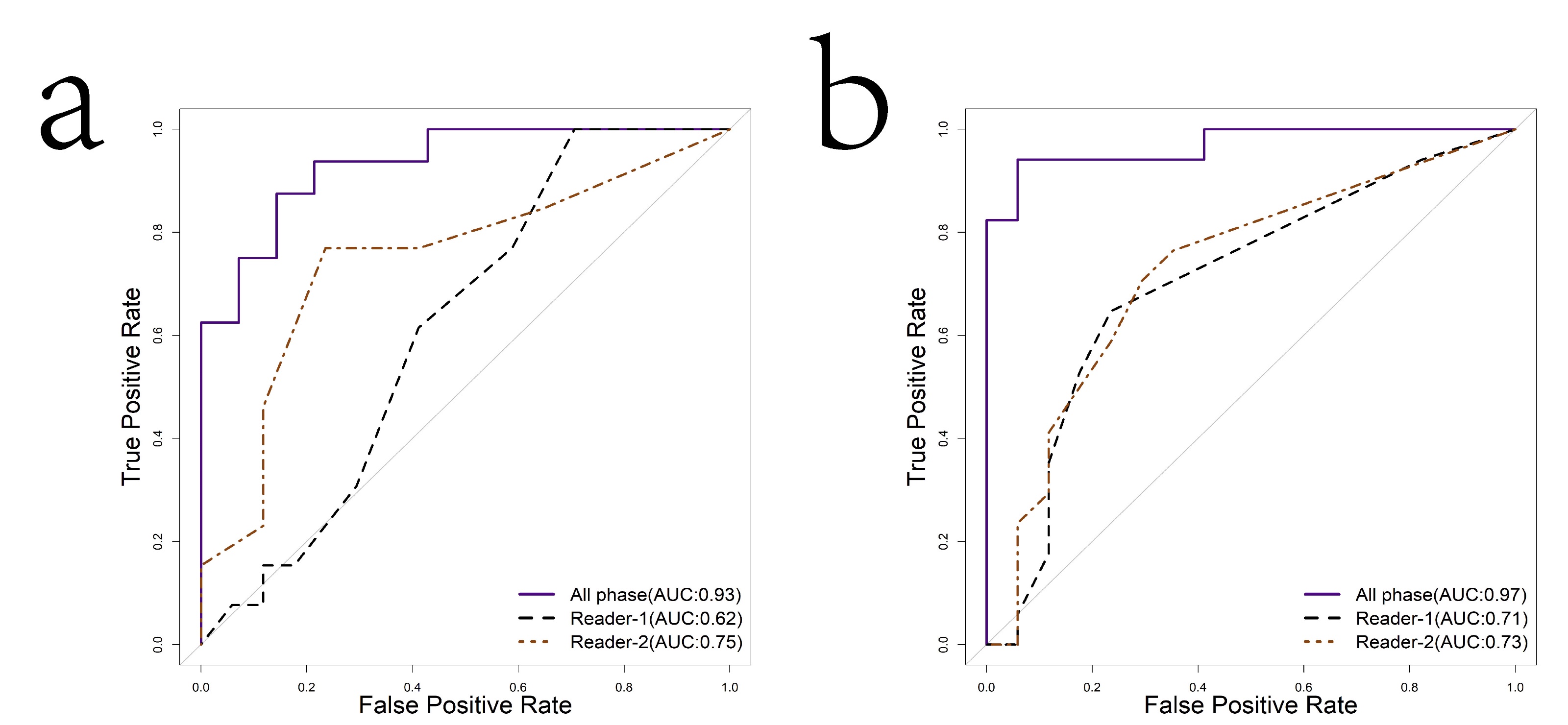


Figure2. In the intra-group classification, AUROC curve was presented for the performance of two experienced radiologists for the reader test on testing set (a). In the inter-group classification, AUROC curve was presented for the performance of two experienced radiologists for the reader test on testing set (b).

Table1. The optimal signature of each model

| Model | Filter | Feature class | Feature | Image phase | Weight |
| --- | --- | --- | --- | --- | --- |
| T1WI | Original | First order | 90Percentile | T1WI | -13.64 |
| (intra-) | Original | GLCM | SumSquares | T1WI | 6.72 |
|  | LoG (σ=2mm) | First order | 10Percentile | T1WI | -5.78 |
|  | LoG (σ=2mm) | First order | Range | T1WI | 10.47 |
|  | LoG (σ=3mm) | First order | 10Percentile | T1WI | -9.02 |
|  | Wavelet (LLH) | GLCM | DifferenceVariance | T1WI | -3.24 |
|  | Wavelet (LLH) | GLSZM | ZoneVariance | T1WI | 6.4 |
|  | Wavelet (HLH) | GLDM | DependenceNonUniformity | T1WI | -11.62 |
|  | Wavelet (LLL) | GLSZM | SizeZoneNonUniformity | T1WI | 9.1 |
| Arterial phase | LoG (σ=2mm) | First order | 10Percentile | Arterial | -2.13 |
| (intra-) | LoG (σ=2mm) | First order | MeanAbsoluteDeviation | Arterial | 1.94 |
|  | LoG (σ=2mm) | First order | Minimum | Arterial | -2.17 |
|  | LoG (σ=2mm) | GLCM | DifferenceAverage | Arterial | 2.09 |
|  | LoG (σ=3mm) | First order | 10Percentile | Arterial | -1.88 |
|  | Wavelet (LLL) | First order | Minimum | Arterial | -1.19 |
| Portal venous phase | Original | GLSZM | SizeZoneNonUniformity | Portal venous | 1.9 |
| (intra-) | LoG (σ=2mm) | GLSZM | ZonePercentage | Portal venous | 2.22 |
|  | LoG (σ=3mm) | First order | 10Percentile | Portal venous | -2.14 |
|  | LoG (σ=3mm) | First order | Median | Portal venous | -2.13 |
|  | LoG (σ=3mm) | First order | Range | Portal venous | 1.98 |
|  | Wavelet (LLL) | GLSZM | SizeZoneNonUniformity | Portal venous | 1.78 |
| Delayed phase (intra-) | LoG (σ=2mm) | First order | Maximum | Delayed | 1.52 |
|  | LoG (σ=3mm) | First order | MeanAbsoluteDeviation | Delayed | 13.59 |
|  | LoG (σ=3mm) | GLSZM | SizeZoneNonUniformity | Delayed | 10.3 |
|  | LoG (σ=3mm) | GLDM | LargeDependenceHighGrayLevelEmphasis | Delayed | -0.68 |
|  | LoG (σ=5mm) | GLCM | DifferenceVariance | Delayed | -2.14 |
|  | LoG (σ=5mm) | GLSZM | GrayLevelNonUniformity | Delayed | -0.6 |
|  | LoG (σ=5mm) | GLSZM | SmallAreaHighGrayLevelEmphasis | Delayed | -4.93 |
|  | Wavelet (LLH) | GLDM | DependenceNonUniformity | Delayed | -3 |
|  | Wavelet (LHL) | First order | Maximum | Delayed | 8.48 |
|  | Wavelet (LHL) | First order | Minimum | Delayed | 8.32 |
|  | Wavelet (LHL) | GLSZM | LargeAreaEmphasis | Delayed | 6.7 |
|  | Wavelet (LHH) | GLSZM | LargeAreaHighGrayLevelEmphasis | Delayed | 2.62 |
|  | Wavelet (LHH) | GLSZM | SizeZoneNonUniformity | Delayed | 7.19 |
|  | Wavelet (LHH) | GLDM | DependenceNonUniformity | Delayed | -5.26 |
|  | Wavelet (HLL) | GLCM | DifferenceAverage | Delayed | 3.49 |
|  | Wavelet (HLL) | GLRLM | LongRunHighGrayLevelEmphasis | Delayed | -3.39 |
|  | Wavelet (HLH) | GLDM | DependenceNonUniformity | Delayed | -3.28 |
|  | Wavelet (HHL) | First order | Range | Delayed | 2.18 |
|  | Wavelet (HHL) | GLSZM | LargeAreaHighGrayLevelEmphasis | Delayed | 6.1 |
|  | Wavelet (HHH) | First order | Variance | Delayed | 3.94 |
|  | Wavelet (HHH) | GLDM | DependenceNonUniformity | Delayed | -5.23 |
|  | Wavelet (LLL) | First order | Minimum | Delayed | -9.41 |
|  | Wavelet (LLL) | GLSZM | GrayLevelNonUniformity | Delayed | -2.3 |
| All-phase | LoG (σ=2mm) | First order | 10Percentile | Arterial | -5.05 |
| (intra-) | LoG (σ=2mm) | First order | Median | Arterial | -5.42 |
|  | LoG (σ=2mm) | GLCM | DifferenceAverage | Arterial | 6.74 |
|  | LoG (σ=4mm) | GLSZM | GrayLevelNonUniformity | Arterial | 5.62 |
|  | LoG (σ=5mm) | GLCM | DifferenceAverage | Arterial | -1.66 |
|  | Wavelet (LLH) | First order | MeanAbsoluteDeviation | Arterial | 1.17 |
|  | Wavelet (LLH) | GLCM | ClusterTendency | Arterial | 0.45 |
|  | Wavelet (LLH) | GLCM | DifferenceAverage | Arterial | -0.02 |
|  | Wavelet (LLH) | GLSZM | ZoneVariance | Arterial | 7.76 |
|  | Wavelet (HLL) | GLDM | LargeDependenceHighGrayLevelEmphasis | Arterial | 11.01 |
|  | Wavelet (LLL) | First order | 10Percentile | Arterial | -1.58 |
|  | Wavelet (LLL) | First order | MeanAbsoluteDeviation | Arterial | 6.82 |
|  | LoG (σ=2mm) | First order | Maximum | Delayed | 7.03 |
|  | LoG (σ=3mm) | First order | MeanAbsoluteDeviation | Delayed | 7.32 |
|  | LoG (σ=4mm) | GLCM | ClusterTendency | Delayed | 6.63 |
|  | Wavelet (LHL) | First order | Maximum | Delayed | 7.96 |
|  | Wavelet (LHL) | GLSZM | LargeAreaLowGrayLevelEmphasis | Delayed | 6.26 |
|  | Wavelet (HHL) | First order | Range | Delayed | 1.87 |
|  | Wavelet (HHH) | GLDM | DependenceNonUniformity | Delayed | -4.79 |
|  | Wavelet (LLL) | First order | Minimum | Delayed | -3.79 |
|  | Original | First order | 90Percentile | T1WI | -4.12 |
|  | LoG (σ=2mm) | First order | 10Percentile | T1WI | -5.59 |
|  | LoG (σ=2mm) | First order | Median | T1WI | -3.22 |
|  | LoG (σ=2mm) | First order | Range | T1WI | 9.66 |
|  | LoG (σ=2mm) | GLSZM | GrayLevelNonUniformity | T1WI | -1.46 |
|  | Wavelet (LLH) | GLCM | ClusterProminence | T1WI | -0.11 |
|  | Wavelet (HLH) | GLRLM | GrayLevelNonUniformityNormalized | T1WI | 3.03 |
|  | Wavelet (HLH) | GLDM | DependenceNonUniformity | T1WI | -5.48 |
|  | LoG (σ=2mm) | First order | Maximum | Portal venous | 2.87 |
|  | LoG (σ=2mm) | GLSZM | ZonePercentage | Portal venous | 3.54 |
|  | Wavelet (LHL) | GLSZM | SizeZoneNonUniformity | Portal venous | 6.23 |
|  | Wavelet (LHH) | GLSZM | ZoneVariance | Portal venous | 6.49 |
|  | Wavelet (LHH) | GLDM | DependenceNonUniformity | Portal venous | -2.38 |
|  | Wavelet (HLL) | GLSZM | GrayLevelNonUniformity | Portal venous | -0.37 |
|  | Wavelet (HLH) | GLCM | ClusterShade | Portal venous | -4.14 |
|  | Wavelet (HHH) | GLDM | DependenceNonUniformity | Portal venous | -4.54 |
| T1WI | Original | First order | 90Percentile | T1WI | -0.65 |
| (inter-) | Original | GLSZM | SizeZoneNonUniformity | T1WI | 1.94 |
|  | Original | GLDM | GrayLevelNonUniformity | T1WI | -1.61 |
|  | LoG (σ=2mm) | First order | Minimum | T1WI | -1.91 |
|  | LoG (σ=2mm) | GLSZM | GrayLevelNonUniformity | T1WI | 1.89 |
|  | LoG (σ=3mm) | First order | Median | T1WI | -1.14 |
|  | LoG (σ=3mm) | First order | RootMeanSquared | T1WI | -1.11 |
|  | LoG (σ=3mm) | GLSZM | GrayLevelNonUniformity | T1WI | 1.82 |
|  | LoG (σ=5mm) | GLDM | DependenceNonUniformity | T1WI | 1.69 |
|  | Wavelet (LLH) | GLSZM | ZoneVariance | T1WI | 0.44 |
|  | Wavelet (LHH) | GLSZM | GrayLevelNonUniformity | T1WI | 1.85 |
|  | Wavelet (HLL) | GLSZM | HighGrayLevelZoneEmphasis | T1WI | 1.75 |
|  | Wavelet (HLL) | GLSZM | SizeZoneNonUniformity | T1WI | 1.87 |
|  | Wavelet (HHL) | GLSZM | SizeZoneNonUniformity | T1WI | 1.51 |
|  | Wavelet (LLL) | GLCM | ClusterShade | T1WI | 0.83 |
|  | Wavelet (LLL) | GLRLM | GrayLevelNonUniformity | T1WI | -1.57 |
| Arterial phase | Original | First order | Median | Arterial | -3.84 |
| (inter-) | Original | GLSZM | GrayLevelNonUniformity | Arterial | 3.71 |
|  | Original | GLSZM | SizeZoneNonUniformity | Arterial | 3.31 |
|  | LoG (σ=2mm) | First order | Minimum | Arterial | -3.86 |
|  | LoG (σ=2mm) | GLCM | DifferenceAverage | Arterial | 3.46 |
|  | LoG (σ=3mm) | First order | Median | Arterial | -2.7 |
|  | LoG (σ=3mm) | GLCM | DifferenceAverage | Arterial | 3.43 |
|  | LoG (σ=3mm) | GLSZM | LargeAreaEmphasis | Arterial | -2.35 |
|  | LoG (σ=4mm) | First order | Median | Arterial | -2.8 |
|  | Wavelet (LHH) | GLCM | SumEntropy | Arterial | -0.71 |
|  | Wavelet (LHH) | GLRLM | RunEntropy | Arterial | -0.97 |
|  | Wavelet (HHL) | First order | 10Percentile | Arterial | -2.13 |
|  | Wavelet (HHH) | GLCM | ClusterProminence | Arterial | 3.16 |
| Portal venous phase | Original | First order | Median | Portal venous | -0.49 |
| (inter-) | Original | GLCM | ClusterShade | Portal venous | 0.95 |
|  | LoG (σ=2mm) | GLSZM | ZonePercentage | Portal venous | 2.29 |
|  | LoG (σ=4mm) | GLSZM | GrayLevelNonUniformity | Portal venous | 1.37 |
|  | LoG (σ=5mm) | GLCM | ClusterTendency | Portal venous | 1.56 |
|  | Wavelet (LLH) | First order | RobustMeanAbsoluteDeviation | Portal venous | 1.69 |
|  | Wavelet (LLH) | GLDM | GrayLevelNonUniformity | Portal venous | -1.03 |
|  | Wavelet (LHH) | GLSZM | ZoneVariance | Portal venous | 0.83 |
|  | Wavelet (HHH) | GLSZM | HighGrayLevelZoneEmphasis | Portal venous | 1.58 |
|  | Wavelet (HHH) | GLSZM | ZoneVariance | Portal venous | 0.99 |
|  | Wavelet (LLL) | GLSZM | SizeZoneNonUniformity | Portal venous | 1.79 |
|  | Wavelet (LLL) | GLDM | DependenceNonUniformity | Portal venous | 1.56 |
| Delayed phase (inter-) | Original | First order | Median | Delayed | -0.71 |
|  | Original | GLDM | GrayLevelNonUniformity | Delayed | -0.97 |
|  | LoG (σ=2mm) | GLCM | Autocorrelation | Delayed | 1.97 |
|  | LoG (σ=3mm) | GLCM | DifferenceAverage | Delayed | 1.56 |
|  | LoG (σ=3mm) | GLDM | LargeDependenceHighGrayLevelEmphasis | Delayed | 1.37 |
|  | Wavelet (LLH) | GLCM | DifferenceAverage | Delayed | 1.34 |
|  | Wavelet (LHH) | GLDM | LargeDependenceHighGrayLevelEmphasis | Delayed | -0.23 |
|  | Wavelet (HLL) | GLSZM | SizeZoneNonUniformity | Delayed | 2.09 |
|  | Wavelet (LLL) | GLCM | ClusterShade | Delayed | 0.79 |
|  | Wavelet (LLL) | GLDM | GrayLevelNonUniformity | Delayed | -1.05 |
| All-phase | Original | First order | Median | Arterial | -11.9 |
| (inter-) | Wavelet (LLH) | GLSZM | ZoneVariance | Arterial | 3.9 |
|  | Wavelet (LHH) | GLCM | SumEntropy | Arterial | -6.2 |
|  | Original | First order | Maximum | Delayed | 7.23 |
|  | LoG (σ=2mm) | GLDM | HighGrayLevelEmphasis | Delayed | 0.15 |
|  | LoG (σ=3mm) | GLSZM | ZoneEntropy | Delayed | -5.19 |
|  | LoG (σ=4mm) | First order | Variance | Delayed | 2.89 |
|  | Wavelet (LLH) | GLCM | DifferenceAverage | Delayed | 2.87 |
|  | Wavelet (HLL) | GLSZM | SizeZoneNonUniformity | Delayed | -1.93 |
|  | Wavelet (LLL) | GLDM | GrayLevelNonUniformity | Delayed | -0.31 |
|  | Original | GLSZM | SizeZoneNonUniformity | T1WI | 0.06 |
|  | Original | GLDM | GrayLevelNonUniformity | T1WI | -4.08 |
|  | Wavelet (LHH) | GLSZM | GrayLevelNonUniformity | T1WI | 2.04 |
|  | Wavelet (HLL) | GLRLM | HighGrayLevelRunEmphasis | T1WI | 0.53 |
|  | Wavelet (HLL) | GLRLM | ShortRunHighGrayLevelEmphasis | T1WI | 0.55 |
|  | Wavelet (HLL) | GLDM | HighGrayLevelEmphasis | T1WI | 0.55 |
|  | Wavelet (LLL) | GLCM | ClusterProminence | T1WI | -7.19 |
|  | LoG (σ=2mm) | GLSZM | ZonePercentage | Portal venous | 0.68 |

Abbreviations: intra-, intra-group classification; inter-, inter-group classification; LoG, Laplacian of Gaussian; GLCM, Gray Level Co-occurrence Matrix; GLDM, Gray Level Dependence Matrix; GLSZM, Gray Level Size Zone Matrix; GLRLM, Gray-Level Run-Length Matrix.

Table2. The numbers, volumes and diameters details of VOIs in each dataset on intra- and inter-group classification

|  | Data set | | | VOI volume  (Mean±SD) mm^3^ | | | VOI diameter  (Mean±SD) mm | | |
| --- | --- | --- | --- | --- | --- | --- | --- | --- | --- |
| Group | \| Training set (%) \|  \| Testing set (%) \| \| --- \| --- \| --- \| | | | \| Training set \|  \| Testing set \| \| --- \| --- \| --- \| | | | \| Training set \|  \| Testing set \| \| --- \| --- \| --- \| | | |
| Intra- | SHCC  n=52 (43%) |  | SHCC  n=16 (13%) | | 537.48±533.52 | 540.61±519.32 | | 8.99±3.61 | 9.40±4.26 |
|  | Non-SHCC  n=40 (33%) |  | Non-SHCC  n=14 (11%) | | 361.99±164.59 | 426.68±186.47 | | 6.24±1.39 | 7.21±2.04 |
| Inter- | SHCC  n=51 (37%) |  | SHCC  n=17 (12.5%) | | 564.45±577.54 | 459.52±330.18 | | 9.08±3.65 | 9.10±4.13 |
|  | Non-SHCC n=53 (38%) |  | Non-SHCC  n=17 (12.5%) | | 510.07±90.68 | 522.92±90.43 | | 6.26±0.53 | 6.40±0.53 |

Abbreviations: Intra-, Intra-group classification; Inter-, Inter-group classification; VOI, volume of interest; SD, standard deviation; SHCC, small hepatocellular carcinoma.
